# Duration-Specific Peak Acceleration Demands During Professional Female Basketball Matches

**DOI:** 10.3389/fspor.2020.00033

**Published:** 2020-04-16

**Authors:** Mareike Roell, Janina Helwig, Albert Gollhofer, Kai Roecker

**Affiliations:** ^1^Institute of Sports and Sports Science (IfSS), University of Freiburg, Freiburg im Breisgau, Germany; ^2^Institute of Applied Health Promotion and Exercise Medicine (IfAG), Furtwangen University, Furtwangen, Germany

**Keywords:** activity profile, external load, indoor team sports, inertial measurement unit, acceleration, basketball

## Abstract

**Purpose:** Describing the most intense periods of match-play is important in player monitoring and the development of specific training programs. The aim of this study was to extract maximum accelerations during basketball match-play and describe those as a function over time durations.

**Methods:** Twelve professional female basketballers were monitored during 13 official matches to calculate acceleration profiles. Moving medians of time durations ranging from 0.3 to 1,800 s were computed to extract peak acceleration and deceleration magnitudes for the resultant (|acc_res_|), horizontal (|acc_hor_|), and vertical (|acc_vert_|) planes. The relationship between peak magnitudes and time durations was modeled by an exponential function. Distinct curve characteristics can be described by the function parameters *scale* and *decrease rate*, which refer to an athlete's ability to perform maximum acceleration intensities over short-time (*scale*) and middle-time intervals (*decrease rate*). Generalized linear mixed-models were calculated to determine plane-specific differences in acceleration and deceleration capacities.

**Results:** Function parameters differed significantly between |acc_res_|, |acc_hor_| and |acc_vert_| [effect size (ES) = 0.33–1.15]. Comparisons within each movement plane revealed significant differences between positive and negative |acc_res_| for the parameters *scale* (ES = 0.34) and *decrease rate* (ES = 0.39). All function parameters differed significantly between |acc_vert_|^+^ and |dec_vert_| (ES = 0.39–0.71). Rank analyses between players revealed significant inter-individual differences for all function parameters in all groups.

**Conclusions:** Modeling peak acceleration magnitudes as a function over log-transformed time durations provides an opportunity to systematically quantify the most intense periods of match-play over short, middle and long time intervals (0.3–1,800 s). Detailed knowledge about these periods may positively contribute to training prescriptions, which intend to prepare players for highest intensities experienced during match-play in order to improve performance and prevent injuries. Derived function parameters allow inter-individual comparisons and provide insights into players' physical capabilities. This study further examines plane-specific intensity demands of professional female basketball, emphasizing the need for coaches to prepare players for maximum decelerations in the vertical plane.

## Introduction

Basketball is characterized as an intermittent, physically demanding sport with frequently-occurring high intensity actions (McInnes et al., 1995; Ben Abdelkrim et al., 2007, 2010). The athleticism and physical fitness of athletes play a major role for success and are important to manage actual game demands (Ziv and Lidor, 2009). In order to maximize performances and minimize the individual risk of injuries through systematic training protocols, the evaluation of the overall workload players are exposed to during matches, is essential. For this purpose the differentiation of workloads into external and internal loads has been established (Impellizzeri et al., 2004, 2005). While the internal load refers to the body's psychophysiological reaction to any external stimulus, the external load aims to quantify the overall physical work of different intensities (Impellizzeri et al., 2019). Even though this concept is frequently applied in various sports, the exact dose-response relationship between both aspects in intermittent team sports has not been clarified yet. Generating new findings that contribute to this problem requires a detailed knowledge of sport-specific external loads, since these naturally determine emerging internal loads.

External match-related demands in basketball are characterized by variable executions of acceleration, deceleration, and sudden changes of direction, interspersed with phases of low intensity activities (Ben Abdelkrim et al., 2007). Activity changes occur frequently over the course of a game, ranging between 579 and 1,750 changes per game in female basketball (Taylor et al., 2017). Considering the short duration of the performed motions [for instance the average duration for sprints is 0.5–2.4 s (Taylor et al., 2017)], acceleration efforts might play a meaningful role in the quantification of locomotor demands. Recently, the mean number of acceleration and deceleration events per quarter for female U18 players was reported to be 159.7 and 153.1, respectively, with mean acceleration efforts lasting 2083.8 ms (Reina et al., 2019). These whole-match variables may be useful for quantification of movement volume during match-play, but they do not account for movement intensity.

With the development of micro-technological devices, intensity measurement becomes feasible by enabling simultaneous measurements of multiple players. Use of GPS units is restricted to outdoor environments, thus inertial measurement units (IMU) serve as an alternative in indoor team sports. Accurate estimations of acceleration in multiple movement planes during sport-specific movements have been shown after adequate data processing (Roell et al., 2019). However, time-ordered visualization of these data remains rather disorganized, which emphasizes the need for a standardized description of actual workloads. For outdoor team sports, attempts at categorization have been carried out using arbitrary velocity thresholds or movement categories (Ben Abdelkrim et al., 2007; Conte et al., 2015). To detect fluctuations in intensity occurring across pre-defined intensity bands or time domains, moving averages have been proposed and applied to multiple team sports (Duthie et al., 2018; Delves et al., 2019; Whitehead et al., 2019). The resulting relationship between peak velocity and moving averages of 1–10 min duration can be described by an exponential decay function (Delaney et al., 2018). Recently, a comparable approach has been proposed, including shortest and longest time intervals ranging from 0.3 to 2,700 s (Roecker et al., 2017). Fitting a mathematical function over these time durations resulted in a sigmoidal relationship between movement speed and time duration, rather than continuing the exponential curve shape of the middle time domains. Resulting curve shapes can be operationalized by distinct function parameters. In soccer, differences between playing positions have been detected relying on this approach (Roecker et al., 2017). In indoor sports like basketball, comparable analysis of the most intense periods of match-play have not been carried out so far.

Analyses in basketball have instead focused on a general quantification of locomotor demands with respect to covered distance, velocity zones, or number of accelerations (Ben Abdelkrim et al., 2007; Puente et al., 2017; Vázquez-Guerrero et al., 2018). Movement categorizations revealed the multi-directional nature of basketball, including horizontal and vertical movements such as sprinting, shuffling, and jumping (Scanlan et al., 2011). It was shown that players most often perform forward-directed movements such as running. Lateral shuffling movements were performed up to 19.8% of playing time at various intensities, whereas jumping movements accounted for 0.6–2.3% for male and female players of different levels (Stojanović et al., 2018). Nonetheless, information about movement intensity with respect to movement planes is limited.

The multi-directional and intermittent character of indoor team sports such as basketball challenges the description of most-intense periods of match-play. However, knowledge about maximum acceleration intensities achieved during competition may provide information about player's physical capacities. By progressively increasing workloads, coaches aim to prepare athletes adequately for actual game demands. Analysis of individualized acceleration profiles will positively contribute to the specificity of training programs. Athletes that have already experienced workloads of maximum intensity during controlled training situations are less likely to suffer an injury, but likely maximize their performance. Therefore, the aim of this study was to model peak acceleration intensities over pre-defined time intervals and to create a match-derived acceleration profile for indoor basketball. As a second goal, differences in acceleration profiles across multiple movement planes were determined.

## Materials and Methods

### Design

IMU data were collected over two consecutive seasons of a professional female basketball team playing in the 2nd (2017/18) and 1st (2018/19) German basketball leagues. All players volunteered to participate in this study and gave written informed consent to the experimental procedure. Approval was granted by the ethics committee of the University of Freiburg (429/18) and was in accordance with the latest revision of the Declaration of Helsinki.

### Subjects

A total of 12 players from the same club participated in this study (20.67 ± 2.69 years, 1.81 ± 0.07 m, 73.87 ± 7.06 kg). Official games of the team following FIBA basketball rules were monitored, resulting in a total of 6 games monitored in the 2017/18 season and 7 games in the 2018/19 season. Datasets were excluded if players participated <5 min in the game. Overall, 117 game observations where recorded, whereas 77 observations were included for data analyses. In the mean athletes had a playing time of 18:18 ± 8:50 min.

### Procedures

For all matches, acceleration values were recorded continuously using 12 IMUs (Catapult S5, Catapult Innovations, Melbourne, Australia) operating at a sampling frequency of 100 Hz. The dimension of the devices are 88 × 50 × 19 mm at a weight of 0.088 kg (Catapult Innovations, Melbourne, Australia). The measurement range of the accelerometer reaches up to ±16 g, of the magnetometer up to 4,800 μT and of the gyroscope up to 2,000°/s. Players were handed the IMU-based devices 60 min before the start of the game prior to the warm-up procedure. Each participant was equipped with one device, which was positioned between the athlete's shoulder blades using a manufacturer-supplied upper body harness. Due to the fit of the harness players were not restricted or hindered in their movement execution. For each player the same device was used for all games in order to eliminate inter-device variability (Boyd et al., 2011; Nicolella et al., 2018).

The activity demands were analyzed throughout the whole game, which in this study is defined as all game-related actions during playing time, stoppages in play (e.g., free throws) and time-outs. Halftime breaks were excluded from analyses.

IMUs require complex data processing, thus the raw accelerometer and gyroscope data were exported from the manufacturer's software (Sprint 5.1.7., Catapult Sports, Melbourne, Australia). If not stated otherwise, further analyses were conducted using customized Matlab scripts (Matlab R2018a, MathWorks, Natick, MA, USA).

A 4th order low-pass digital zero-lag Butterworth filter was applied to reduce noise from the raw accelerometer data. According to a residual analysis an optimal cut-off frequency of 8 Hz was chosen (Winter, 2009). To exclude phase-shift dual pass filtering and a correction of the cut-off frequency to 9.97 Hz was applied (Winter, 2009). The devices' attitude was corrected through application of a previously-validated complementary filter algorithm on the accelerometer and gyroscope data (compiled and edited with C++) (Valenti et al., 2015; Roell et al., 2018, 2019). For higher congruence between IMU data and actual body movements, attitude-corrected accelerometer data were resampled to 5 Hz by averaging (Roell et al., 2019). Magnitudes of the 3D resultant acceleration vector (|acc_res_|), the horizontal vector (|acc_hor_|), and the vertical acceleration vector (|acc_vert_|) were calculated according to the following formulas.

(1) $$\begin{array}{lll} \left|acc_{res}\right| & = & \sqrt{x^{2}+y^{2}+z^{2}} \end{array}$$

(2) $$\begin{array}{lll} \left|acc_{hor}\right| & = & \sqrt{x^{2}+y^{2}} \end{array}$$

(3) $$\begin{array}{lll} \left|acc_{vert}\right| & = & \sqrt{z^{2}} \end{array}$$

To discriminate between acceleration and deceleration efforts, continuous vectors were calculated for |acc_res_|^+^, |dec_res_|, |acc_hor_|^+^, |dec_hor_|, |acc_vert_|^+^, and |dec_vert_| including only positive or negative acceleration values with respect to anterior-posterior and vertical movement directions. The sign of the medio-lateral acceleration data was not considered for these calculations as this would rather represent information about movements to the left and right direction than information about acceleration or deceleration.

Moving medians for time durations (t_dur_) ranging from 0.3 to 1,800 s were applied. Due to their stability against outliers, medians were preferred over moving averages (Dixon, 1953). The peak median for each time interval was detected and used for the fitting procedure. Prior to the fitting procedure, t_dur_ was log-transformed for better scaling of the shortest and longest time intervals. Based on these values, profiles were fitted for each player and each game by a two-parameter exponential algorithm:

(4) $$\begin{array}{l} |acc|\left(t_{dur}\right)=a\cdot e^{b\cdot \log\left(t_{dur}\right)} \end{array}$$

**Figure 1:**
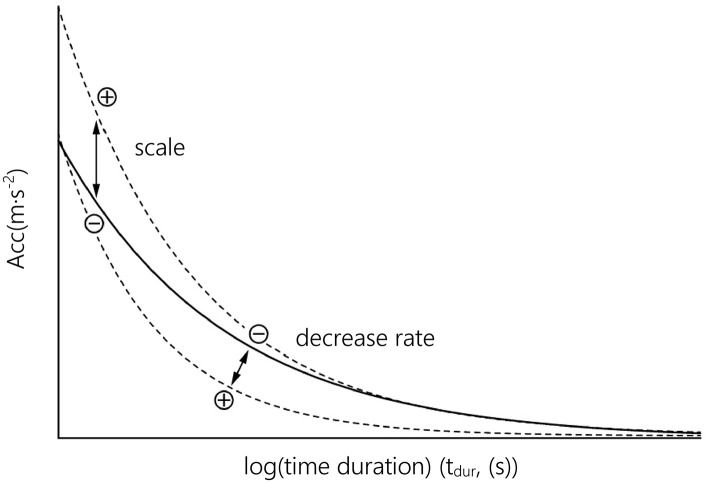
Function parameters (*scale, decrease rate*) describing the function's curve shape. The solid black line represents the original fitting for peak median intensities. Dashed lines show the effect of change on either *scale* or *decrease rate*.

It is assumed that each function parameter represents physical capacities. *Scale* (a, Figure 1) contains information about the curve's inclination. Higher scaling factors induce a more declined progress of the curve and can be associated with the ability of players to maintain the highest intensities in an intermittent manner. The curve's *decrease rate* (b) quantifies the decrement in acceleration with increasing time intervals and is presumably associated with the amount of fatigue experienced by an athlete. Additionally, we calculated the predicted acceleration value at a time duration of 0.5 s to investigate the achieved peak acceleration (*acc*_*max*_).

### Statistical Analyses

Statistical analyses, model fittings and evaluations were performed using JMP version 14.2.0 (SAS Institute Inc., Cary, NC, USA). Unless otherwise indicated, data are presented as means ± SD with statistical significance set at *p* ≤ 0.05. Shapiro-Wilk tests revealed heteroscedastic data sets. Spearman's ρ was calculated to determine possible linear relationships between function parameters.

As repeated measures occurred in group comparisons, a general linear mixed model with “participant,” “game,” and “playing time” as random factors was applied to determine significant differences of the function parameters. As fixed effects for the mixed-model analyses factors “movement plane” (|acc_res_|, |acc_hor_|, |acc_vert_|), “algebraic signs” (acceleration/deceleration) and their interaction effect were defined. To test pairwise comparisons, a Steel-Dwass all-pairs test on ranks was applied for group comparisons. Where significant differences occurred for random factors, Van der Waerden tests based on rank scores were calculated *post-hoc*.

## Results

**Figure 2:**
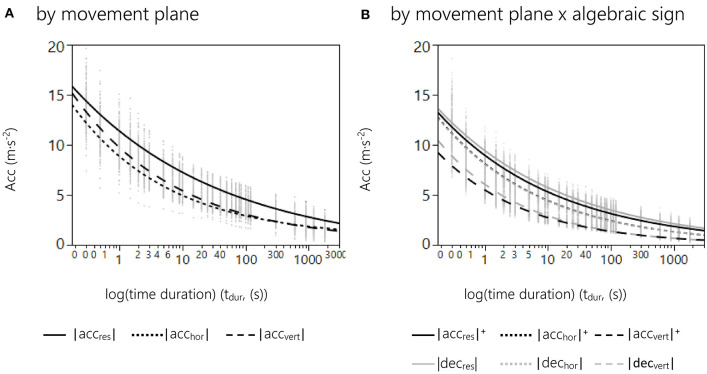
**(A)** Curve fitting results of the 2 parameter exponential model grouped by movement plane. The graphs represent the relationship between peak median intensities over log-transformed time domains for |acc_res_| (solid), |acc_hor_| (dotted), and |acc_vert_| (dashed). Gray dots represent peak median accelerations for time durations from 0.3 to 1,800 s for each game and player. **(B)** Curve fitting results of the 2 parameter exponential model grouped by movement plane × algebraic sign. The graphs represent the relationship between peak median intensities over log-transformed time domains for |acc_res_|^+^ (black solid), |acc_res_|^−^ (gray solid), |acc_hor_|^+^ (black dotted), |acc_hor_|^−^ (gray dotted), |acc_vert_|^+^ (black dashed), and |acc_vert_|^−^ (gray dashed). Gray dots represent peak median accelerations for the time durations from 0.3 to 1,800 s for each game and player.

Our results show that modeling the relationship between acceleration and time duration in female basketball can best be described by an exponential decay function. Since this function was present for each subject, it can be stated that the presented modeling follows a fundamental regularity. Resulting curve shapes for fittings by *movement plane* and *movement plane* × *algebraic sign* are displayed in Figures 2A,B.

For evaluation of the fitting model, several parameters were calculated and are reported in Table 1.

**Table 1:** Evaluation of the fitting model representing the relationship between peak median acceleration (y) and time duration (t_dur_; x) for every player and game tested in the study.

	**AICc**	**SSE**	**MSE**	**RMSE**	**r^**2**^**
|acc_res_|	35.69 ± 17.50	5.43 ± 3.29	0.20 ± 0.12	0.43 ± 0.13	0.98 ± 0.01
|acc_hor_|	29.06 ± 17.67	4.40 ± 3.05	0.16 ± 0.11	0.38 ± 0.12	0.98 ± 0.01
|acc_vert_|	26.82 ± 17.95	4.15 ± 3.37	0.15 ± 0.12	0.37 ± 0.13	0.99 ± 0.01
|acc_res_|^+^	38.60 ± 17.75	6.02 ± 3.58	0.22 ± 0.13	0.45 ± 0.14	0.97 ± 0.02
|dec_res_|	26.69 ± 18.07	4.07 ± 2.96	0.15 ± 0.11	0.37 ± 0.12	0.98 ± 0.01
|acc_hor_|^+^	28.79 ± 19.20	4.38 ± 2.61	0.16 ± 0.10	0.38 ± 0.12	0.98 ± 0.01
|dec_hor_|	25.30 ± 16.71	3.71 ± 1.94	0.14 ± 0.07	0.36 ± 0.10	0.98 ± 0.01
|acc_vert_|^+^	12.28 ± 17.86	2.43 ± 1.39	0.09 ± 0.05	0.29 ± 0.09	0.97 ± 0.01
|dec_vert_|	38.20 ± 12.44	5.45 ± 2.31	0.20 ± 0.09	0.44 ± 0.09	0.95 ± 0.02
Mean	29.05 ± 17.24	4.45 ± 2.72	0.16 ± 0.10	0.39 ± 0.12	0.98 ± 0.01

*AICc, Akaike's Information Criterion; SSE, residual sum of squares error; MSE, mean squared error; RMSE, standard deviation of the residual error; r^2^, coefficient of determination*.

To evaluate the independency of the function parameters correlation analyses were calculated between the parameters *scale, decrease rate*, and *acc*_*max*_. The results point out a significant large positive correlation between parameters *scale* and *acc*_*max*_ (ρ = 0.99, *p* < 0.0001). Between parameters *scale* and *decrease rate* a significant moderate positive correlation was observed (ρ = 0.33, *p* < 0.0001). A small positive correlation coefficient was observed between the parameters *acc*_*max*_ and *decrease rate* (ρ = 0.17, *p* = 0.001).

The function parameters for each movement plane are given in Table 2. For all function parameters differences for the fixed factor *movement planes* were observed as the following. In general, significant differences were found between |acc_res_|, |acc_hor_|, and |acc_vert_| for *scale, decrease rate* and *acc*_*max*_ (*p* < 0.0001). In addition, the random factor *player* had a significant effect on *scale* (*p* = 0.03), *decrease rate* (*p* = 0.04) and *acc*_*max*_ (*p* = 0.03). *Post-hoc* tests revealed significant differences between single movement planes. The function parameters regarding *scale* (*p* < 0.0001, r = 1.15)*, decrease rate* (*p* < 0.0001, r = 1.07), and *acc*_*max*_ (*p* < 0.0001, r = 1.03) differed significantly between |acc_res_| and |acc_hor_|. This results in a more flattened curve shape for |acc_res_| than for |acc_hor_|. All function parameters differed significantly between |acc_res_| and |acc_vert_| (*scale* (*p* < 0.0001, r = 0.95)*, decrease rate* (*p* = 0.009, r = 1.15), and *acc*_*max*_ (*p* < 0.0001, r = 0.77). Comparisons between |acc_hor_| and |acc_vert_| revealed significant differences regarding *scale* (*p* < 0.0001, r = 0.56), *decrease rate* (*p* < 0.0001, r = 0.33), and *acc*_*max*_ (*p* < 0.0001, r = 0.57).

**Table 2:** Parameters characterizing the exponential curve shape of the relationship between peak median accelerations and time durations (t_dur_) ordered by the groups *movement planes* and *movement planes* × *algebraic sign*.

	**Scale**	**Decrease rate**	**acc_**max**_**
**Movement planes**			
|acc_res_|	11.46 ± 1.08^A^^,^^B^	−0.20 ± 0.02^A^^,^^B^	13.16 ± 1.33^A^^,^^B^
|acc_hor_|	8.97 ± 0.94^A^^,^^C^	−0.24 ± 0.03^A^^,^^C^	10.63 ± 1.27^A^^,^^C^
|acc_vert_|	9.81 ± 0.98^B^^,^^C^	−0.25 ± 0.02^B^^,^^C^	11.71 ± 1.21^B^^,^^C^
**Movement planes** **×** **algebraic sign**			
|acc_res_|^+^	8.93 ± 0.88^*^	−0.23 ± 0.02^*^	10.47 ± 1.12
|dec_res_|	9.40 ± 0.90^*^	−0.22 ± 0.02^*^	10.92 ± 1.11
|acc_hor_|^+^	8.10 ± 0.89	−0.26 ± 0.03	9.73 ± 1.18
|dec_hor_|	8.05 ± 0.82	−0.26 ± 0.03	9.67 ± 1.12
|acc_vert_|^+^	5.47 ± 0.58^*^	−0.30 ± 0.03^*^	6.76 ± 0.77^*^
|dec_vert_|	6.03 ± 0.50^*^	−0.32 ± 0.03^*^	7.54 ± 0.58^*^

*Results are reported as mean values ± SD*.

A *Significant difference between |acc_res_| and |acc_hor_|*.

B *Significant difference between |acc_res_| and |acc_vert_|*.

C *Significant difference between |acc_hor_| and |acc_vert_|*.

* *Significant difference between algebraic signs within one movement plane*.

To determine differences between acceleration and deceleration efforts within each movement planes, comparisons for the combined factor *movement planes* × *algebraic signs* were calculated. Within each movement plane, there were significant differences between acceleration and deceleration profiles for all three parameters (*p* < 0.0001; Figure 2). In addition, the random factor *player* had a significant effect on *scale* (*p* = 0.03), *decrease rate* (*p* = 0.04), and *acc*_*max*_ (*p* = 0.03). Within movement planes, *post-hoc* tests revealed significant differences between |acc_res_|^+^ and |dec_res_| for *scale* (*p* < 0.0001, r = 0.34) and *decrease rate* (*p* = 0.009, r = 0.39). There were no significant differences between |acc_hor_|^+^ and |dec_hor_|. The largest differences were seen between |acc_vert_|^+^ and |dec_vert_| regarding *scale* (*p* < 0.0001, r = 0.71), *decrease rate* (*p* = 0.007, r = 0.39), and *acc*_*max*_ (*p* < 0.0001, r = 0.79).

**Figure 3:**
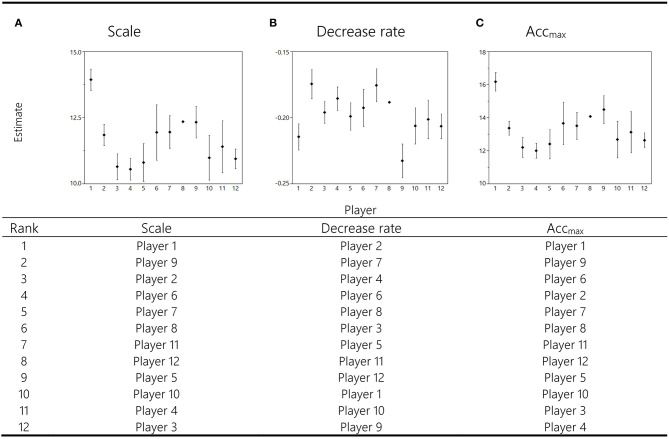
Preliminary longitudinal results representing the |acc_res_| mean value ± SD for each function parameter across all matches for each player (x-axes). Estimated value for curve function parameters (*decrease rate, scale*, and *acc*_*max*_) are shown on the respective y-axes. The table below shows according results for longitudinal comparisons between players based on ranks. Lower rank numbers indicate better performances with respect to *scale, decrease rate*, and *acc*_*max*_.

*Post-hoc* analyses of ranks were based on mean values of *acc*_*max*_, *scale* and *decrease rate* for each player over all game participations. Analyses of ranks for the factor *player* revealed significant differences for *scale* (*p* ≤ 0.0001, v = 0.25), *decrease rate* (*p* ≤ 0.0001, v = 0.22), and *acc*_*max*_ (*p* ≤ 0.0001, v = 0.27) in all groups (*movement plane [*×*algebraic sign]*). Exemplary, parameter estimates for |acc_res_| are shown in Figure 3. Ranks were assigned to each player: note that lower rank numbers indicate better performances.

## Discussion

The aim of this study was to describe the relationship between accelerations and time duration of movement demands for professional female basketball players during indoor basketball matches. Our results show that accelerations in basketball can be described uniformly by an exponential relationship to log-transformed time intervals, characterized by the parameters *scale, decrease rate*, and *acc*_*max*_. This curve shape has been true for data of every player participating in the study. Comparisons between movement planes suggest a different contribution of horizontal and vertical components to the overall resultant acceleration, as well as differences between acceleration and deceleration loads within movement planes.

In general, the fitting quality of the exponential function was comparable between all parameters. According to Aikaike's Information Criterion highest fitting qualities appeared for the vertical plane in comparison to the three-dimensional and horizontal dimension. Considering the reduced accuracy of the IMU-based device in the horizontal plane (Roell et al., 2019), it can be assumed that measurement errors affect the measurement quality of |acc_res_| and |acc_hor_|. Consequently, increased measurement bias reduces the fitting quality in these movement planes. Further improvements are expected due to the ongoing technical development and improvement of the devices accuracy.

Regarding the function parameters, large positive correlations between the *scale* and *acc*_*max*_ indicate that both parameters describe equivalent physical capacities and underlying physiological determinants. We assume that both parameters refer to the ability of an athlete to generate and tolerate maximum acceleration efforts during shortest time intervals, which is associated with aspects of neuromuscular activation (Mero and Komi, 1987; Little and Williams, 2005). A large variance between the function parameters *scale* and *decrease rate* indicates that both parameters represent different physical capacities as well as neuromuscular and metabolic determinants. Most likely, the parameter *decrease rate* contains information about the amount of fatigue, which induces a reduction of intensity with increasing time intervals. In intermittent team sports fatigue is typically associated with the execution of repeated acceleration sequences, which describe the execution of at least three repeated maximal or near maximal high intensity accelerations interspersed with short periods of recovery (Barberó-Álvarez et al., 2014). A reduced capacity to perform repeated high intensity efforts is associated with a substantial reduction of performance (Mendez-Villanueva et al., 2008). On a physiological level, both neuromuscular as well as metabolic factors modulate the amount and severity of fatigue (Mendez-Villanueva et al., 2008). Athletes, whose acceleration profile is characterized by a small *decrease rate* are suspected to maintain high intensities during repeated acceleration efforts. This may indicate an increased metabolic capacity with respect to involved metabolic pathways as well as an increased tolerance against fatigue-induced neuromuscular alterations. However, the relationship between the parameter *decrease rate* and an athlete's repeated acceleration ability or its physiological determinants, has to be examined in a future content validation of the presented acceleration profiles.

Describing the most intense periods as a function of time has been proposed to account for fluctuations of intensities over the course of a game (Delaney et al., 2018). This study attempted to structure the chaotic nature of intensities in intermittent team sports such as basketball by a moving median technique. Using this approach, a decrease of peak acceleration magnitudes with increasing time intervals has been observed. Describing this relationship by a mathematical model leads to function parameters, which represent distinct physical capacities of players. Comparable analyses have been carried out for locomotor variables in multiple team sports by a moving average method (Roecker et al., 2017; Duthie et al., 2018; Delves et al., 2019; Whitehead et al., 2019). Delaney and colleagues modeled the function of velocity efforts over 1–10 min time intervals in soccer as an exponential power law function (2018). In contrast, including both shortest and longest time intervals resulted in a sigmoidal curve shape for velocity data in soccer (Roecker et al., 2017). Comparing both approaches, it seems that the exponential curve shape is appropriate to describe peak velocities over middle time durations but does not account for time intervals <1 min and >10 min. According to Roecker and colleagues, these end ranges are mathematically described by an upper and a lower asymptote, respectively, which was seen for velocity data (Roecker et al., 2017). Analysis of match-derived acceleration data in rugby and soccer has confirmed the exponential curve shape over middle time intervals. Following the approach by Roecker and colleagues, this is the first study to include shortest and longest time durations to determine the relationship between acceleration intensity and time duration. In contrast to the velocity data, our acceleration data did not show a sigmoidal curve shape. The exponential curve shape, which has been established for middle time durations, seems to be continued for time durations <1 min, where an exponential increase with decreasing time durations can be seen. Still, including shortest time intervals prior to the fitting procedure allows for more accurate estimations of intensity demands for time durations <1 min. These time domains seem to be of special interest for basketball, as it was shown that acceleration efforts occur every 7.1 ± 1.7 s on average for experienced male players (Puente et al., 2017). These efforts last about 2 s, which substantiates the importance of accurate acceleration estimates during short-time intervals. Time durations <1 min should thereby be considered for fitting procedures.

Considering the sigmoidal curve found for velocity profiles in soccer, the translation of acceleration into velocity for the shortest time possible seems to be hindered as players reach a plateau of maximum possible velocity (Roecker et al., 2017). This could be explained following basic biomechanical rules, such as requiring a certain amount of time to overcome inertia and induce displacement. It is known from sprinting that elite sprinters need about 6 s to reach maximum velocity during a 100 m race, assuming maximal acceleration (Bissas et al., 2018). It is likely that the highest velocities in intermittent team sports cannot be achieved during ultra-short time durations (<1 s) despite an increasing acceleration. Yet, execution of these acceleration efforts likely leads to high energetic costs, which emphasizes the physiological relevance of acceleration-based activity profiles. However, simultaneously-recorded velocity and acceleration data do not yet exist within one single sport, which would be necessary to substantiate these assumptions.

Longitudinal analyses of our data revealed that the parameters of each function remained reproducible for each player over the course of all monitored games (Figure 3). This indicates that the described parameters are characteristic for each player in the long term. A unique set of parameters enables us to determine the individual capacities of players. These data allow for justified and profound comparisons between players or playing positions. Our results indicated inter-individual differences for all parameters, suggesting different capacities for performing peak intensities and repeated high-intensity efforts. Players can clearly be discriminated by these parameters and ranked (Figure 3). However, due to the limited number of data sets, these assumptions remain preliminary; but provide promising indications for the future practical application of this approach.

Our study not only determines the general relationship between acceleration and time duration, but also allows a deeper understanding of the movement components in professional female basketball. The results indicate that both horizontal and vertical acceleration contribute to basketball-specific motion characteristics, although in different proportions. Significant differences were seen between |acc_res_|, |acc_hor_|, and |acc_vert_|. We found that |acc_res_| represents the overall acceleration and deceleration load imposed on the athlete and is described by a rather flattened exponential function (Figure 2A). This might be explained by the opposing contributions from the horizontal and vertical components. Quick horizontal movements (sprints, shuffling) induce only low vertical acceleration, whereas large vertical acceleration (jumps, landings) results in rather low horizontal acceleration. Regarding the magnitude of |acc_res_|, low values in one component are likely balanced by the other. Hence, looking only at the |acc_res_| might result in a blurred picture about actual workloads. Analyses of multiple movement planes are needed for expedient training recommendations.

Comparisons between the horizontal and vertical component revealed higher *acc*_*max*_ over short time intervals for the vertical plane. This is probably due to the intense bouts of acceleration occurring during jumps and landings. For middle-duration intervals, the curve shapes indicate that higher intensities are experienced in the vertical plane. Considering the number of movement actions performed throughout basketball matches, it is suggested that players perform more horizontal-dominated movements such as sprinting and shuffling compared to vertical jumps (McInnes et al., 1995; Ben Abdelkrim et al., 2007; Scanlan et al., 2011). Despite the higher intensity of jumping movements, their reduced volume might prevent players from suffering fatigue in this movement plane, which enables them to maintain higher intensities compared to the horizontal plane. Additionally, the large decelerations experienced during landing contribute to |acc_vert_| and likely result in overall higher values.

Within each movement plane, differences were apparent between acceleration- and deceleration-specific curve parameters. A larger decrement of intensity with increasing time intervals was seen for resultant deceleration (|dec_res_|) compared to acceleration intensities (|acc_res_|^+^). Similar results were found in the horizontal and vertical planes, respectively. Differences are limited to short and middle durations only, which can be assigned to the repeated high-intensity actions. The ability to perform the highest accelerations during these actions seems to be impaired compared to decelerations, which might be explained by the larger energy costs required for acceleration. It was shown that repeated high-intensity efforts lead to rapidly increasing fatigue (Mendez-Villanueva et al., 2008; Girard et al., 2011). Multiple causes contribute to this phenomenon, ranging from neural factors to the accumulation of metabolites. With respect to acceleration movements, the greater metabolic demands might primarily explain the overall lower intensity in middle duration intervals. However, peak decelerations within these time intervals remain high due to the summation of intended decelerations (changes of direction) and “accompanying” decelerations occurring during landings and tackles. Decelerations are associated with higher mechanical loads (Harper and Kiely, 2018), which induce greater damage on soft-tissue structures (Dalen et al., 2016; Gastin et al., 2019). Consequently, high frequencies of decelerations are associated with decrements in neuromuscular performance and indicate post-match muscle damage (Howatson and Milak, 2009; Gastin et al., 2019). It is therefore essential to prepare players for these load intensities under controlled training conditions.

For the vertical plane, a higher *acc*_*max*_ was observed for deceleration compared to acceleration values (Table 2). The differences probably result from greater intensities experienced during landings compared to jumping itself. This consequently induces increased ground reaction forces (Ortega et al., 2010), which produce large stresses on the passive structures of lower limbs and are associated with an increased risk of injury (Dufek and Bates, 1991). Even though initial vertical deceleration is higher, a more rapid decrement of intensity over increasing time duration is seen compared to acceleration. Cormack and colleagues examined the influence of neuromuscular fatigue on the proportion of plane-specific acceleration to the overall acceleration load during intermittent running (Cormack et al., 2013). Fatigue-induced reductions of acceleration during running occurred in the vertical plane, indicating a direct impairment of acceleration and deceleration capacities. A differentiation in positive and negative vertical acceleration has not been considered in their study. Our results suggest that the reduction of intensity is more prominent for vertical deceleration than acceleration. This needs to be considered for individualized load management. To reduce these peak intensities, coaches should include movements associated with neuromuscular stress in their training and specifically focus on the vertical planea.

### Limitations

The validity of the IMUs has to be mentioned as a limitation of this study. Although the data processing system has previously been validated during team sport-specific movements against a 3D motion analysis system (Roell et al., 2018, 2019), reduced accuracy has been reported for peak values of the horizontal resultant acceleration. Using the median as a robust variable against outliers for every time interval in this study is expected to account for this error and minimize inaccuracies in the horizontal plane.

Regardless of the movement plane, higher errors were reported for the highest intensity values (Wundersitz et al., 2015; Roell et al., 2019), which has to be considered for values in the ultra-short time intervals. Inaccuracies can be limited by the applied resampling procedure. Further, the function's model is based on the entirety of median peak values, which stabilizes the model by incorporating accurate low-intensity values. Using estimated *acc*_*max*_ is presumed to be of higher accuracy than measured single peak values.

### Practical Applications

According to our results, peak accelerations performed by professional female players during indoor basketball match-play follow an explicit and reproducible curve shape over distinct time intervals. An exponential decrement of peak intensities with increasing time intervals has been determined for all data sets of match participation regardless of the player. This indicates an inter- and intra-individual regularity for the interrelationship between acceleration and time domains in professional female basketball. It is to expect that the function parameters *scale* and *decrease rate* represent individual player characteristics. Since the variation of these parameters appears to be stable over the course of multiple games, tactical or game-related aspects seem to affect the physical abilities and underlying physiological correlates only to a limited extent. Analyses on ranks showed that players can be distinguished based on their different physical performance levels. Assuming that over the course of a season each player shows at least once her maximum acceleration capacity per time interval, fundamental statements about individual physical capacities can be done. Thereby, the presented modeling of acceleration-related activity profiles allows characterizing players and evaluating performance without the need for an additional laboratory or field assessment. However, further investigations examining the content validity of the presented acceleration profiles are required to support these assumptions. Knowledge about movement intensities experienced during official games further contributes to the development of individualized training programs. Training drills of variable duration can be designed relying on the relative values of predicted match intensities. In contrast, training demands can be interpreted in relation to these peak values. The described modeling facilitates the work of coaches and improves training processes as it enables quantification of external workloads during intermittent sports like basketball.

### Conclusion

Modeling the resultant, horizontal and vertical acceleration magnitudes as a function of time duration provides a novel opportunity to determine peak acceleration intensities in individual player performances, and allows for standardized and comparable analysis within and between players. A 2-parameter exponential model with distinct parameters (*scale, decrease rate*) can be established. This gives insights into the actual intensity demands during indoor basketball match-play and likely provides information about the underlying physiology. The proposed modeling enables justified discrimination of a single player's physical capacities and provides new insights into actual intensity demands of professional female basketball.

Differences in locomotor demands exist between single movement planes. Discriminant analyses between vertical and horizontal movement planes should be carried out rather than relying on the overall resultant acceleration alone. More specifically, players should be well-prepared for the highest deceleration loadings in the vertical plane.

It has to be expected that extraneous factors that are known to affect performance possibly have an influence on the function parameters. Since the presented acceleration profiles enable to carry out performance diagnostics using in-game data rather than laboratory data, this approach represents a new possibility to detect and understand these factors. Thereby, future research should aim to quantify the impact that extraneous factors, such as match period or playing position, have on acceleration-related performances in realistic game scenarios. In order to understand the underlying physiology of the acceleration profiles future research needs to determine these factors and evaluate the profiles' content validity on a neuromuscular and metabolic level.

## Data Availability Statement

The datasets generated for this study are available on request to the corresponding author.

## Ethics Statement

The studies involving human participants were reviewed and approved by the ethics committee of the University of Freiburg (429/18) and was in accordance with the latest revision of the Declaration of Helsinki. Written informed consent to participate in this study was provided by the participants' legal guardian/next of kin.

## Author Contributions

MR, AG, and KR designed this study. Methodology was planned by MR and KR. MR collected the data. MR, JH, and KR analyzed and interpreted the data. AG provided funding acquisition and resources. MR drafted the manuscript. All authors revised the manuscript and approved the final version to be published.

### Conflict of Interest

KR is scientific consultant for Adidas and is owner of a software company (ergonizer.com) for performance diagnostics. MR receives research grants of Adidas Inc. (Portland, US). AG is scientific consultant for Adidas. This did not play any additional role in the study design, data collection and analysis, decision to publish, or preparation of the manuscript. The remaining author declares that the research was conducted in the absence of any commercial or financial relationships that could be construed as a potential conflict of interest.

## References

[B1] Barberó-Álvarez J. C. Boullosa D. Nakamura F. Y. Andrín G. Weston M. (2014). Repeated acceleration ability (RAA): a new concept with reference to top-level field and assistant soccer referees. Asian J. Sports Med. 5, 63–66. 10.5812/asjsm.34235 24868433 PMC4009089

[B2] Ben Abdelkrim N. Castagna C. Jabri I. Battikh T. El Fazaa S. El Ati J. (2010). Activity profile and physiological requirements of junior elite basketball players in relation to aerobic-anaerobic fitness. J. Strength Condition. Res. 24, 2330–2342. 10.1519/JSC.0b013e3181e381c1 20802281

[B3] Ben Abdelkrim N. El Fazaa S. El Ati J. (2007). Time-motion analysis and physiological data of elite under-19-year-old basketball players during competition. Br. J. Sports Med. 41, 69–75; discussion: 75. 10.1136/bjsm.2006.032318 17138630 PMC2658931

[B4] Bissas A. Walker J. Tucker C. Paradisis G. (2018). Biomechanical Report for the IAAF World Championships London 2017: 100m Women's. Available online at: https://www.worldathletics.org/about-iaaf/documents/research-centre (accessed March 31, 2020).

[B5] Boyd L. J. Ball K. C. Aughey R. J. (2011). The reliability of MinimaxX accelerometers for measuring physical activity in Australian football. Int. J. Sports Physiol. Perform. 6, 311–321. 10.1123/ijspp.6.3.311 21911857

[B6] Conte D. Favero T. G. Lupo C. Francioni F. M. Capranica L. Tessitore A. (2015). Time-motion analysis of Italian elite women's basketball games: individual and team analyses. J. Strength Condition. Res. 29, 144–150. 10.1519/JSC.0000000000000633 25051006

[B7] Cormack S. J. Mooney M. G. Morgan W. McGuigan M. R. (2013). Influence of neuromuscular fatigue on accelerometer load in elite Australian football players. Int. J. Sports Physiol. Perform. 8, 373–378. 10.1123/ijspp.8.4.373 23170747

[B8] Dalen T. Ingebrigtsen J. Ettema G. Hjelde G. H. Wisløff U. (2016). Player load, acceleration, and deceleration during forty-five competitive matches of elite soccer. J. Strength Condition. Res. 30, 351–359. 10.1519/JSC.0000000000001063 26057190

[B9] Delaney J. A. Thornton H. R. Rowell A. E. Dascombe B. J. Aughey R. J. Duthie G. M. (2018). Modelling the decrement in running intensity within professional soccer players. Sci. Med. Football 2, 86–92. 10.1080/24733938.2017.1383623

[B10] Delves R. I. M. Bahnisch J. Ball K. Duthie G. M. (2019). Quantifying mean peak running intensities in Elite Field Hockey. J. Strength Condition. Res. 10.1519/JSC.0000000000003162. [Epud ahead of print]. 31045755

[B11] Dixon W. J. (1953). Processing data for outliers. Biometrics 9, 74–89. 10.2307/3001634

[B12] Dufek J. S. Bates B. T. (1991). Biomechanical factors associated with injury during landing in jump sports. Sports Med. 12, 326–337. 10.2165/00007256-199112050-00005 1763250

[B13] Duthie G. M. Thornton H. R. Delaney J. A. Connolly D. R. Serpiello F. R. (2018). Running intensities in elite youth soccer by age and position. J. Strength Condition. Res. 32, 2918–2924. 10.1519/JSC.0000000000002728 29985216

[B14] Gastin P. B. Hunkin S. L. Fahrner B. Robertson S. (2019). Deceleration, acceleration, and impacts are strong contributors to muscle damage in professional Australian Football. J. Strength Condition. Res. 33, 3374–3383. 10.1519/JSC.0000000000003023 30694964

[B15] Girard O. Mendez-Villanueva A. Bishop D. (2011). Repeated-sprint ability - part I: factors contributing to fatigue. Sports Med. 41, 673–694. 10.2165/11590550-000000000-00000 21780851

[B16] Harper D. J. Kiely J. (2018). Damaging nature of decelerations: do we adequately prepare players? BMJ Open Sport Exerc. Med. 4:e000379. 10.1136/bmjsem-2018-000379 30112183 PMC6089312

[B17] Howatson G. Milak A. (2009). Exercise-induced muscle damage following a bout of sport specific repeated sprints. J. Strength Condition. Res. 23, 2419–2424. 10.1519/JSC.0b013e3181bac52e 19826279

[B18] Impellizzeri F. M. Marcora S. M. Coutts A. J. (2019). Internal and external training load: 15 years on. Int. J. Sports Physiol. Perform. 14, 270–273. 10.1123/ijspp.2018-0935 30614348

[B19] Impellizzeri F. M. Rampinini E. Coutts A. J. Sassi A. Marcora S. M. (2004). Use of RPE-based training load in soccer. Med. Sci. Sports Exerc. 36, 1042–1047. 10.1249/01.MSS.0000128199.23901.2F 15179175

[B20] Impellizzeri F. M. Rampinini E. Marcora S. M. (2005). Physiological assessment of aerobic training in soccer. J. Sports Sci. 23, 583–592. 10.1080/02640410400021278 16195007

[B21] Little T. Williams A. G. (2005). Specificity of acceleration, maximum speed, and agility in professional soccer players. J. Strength Condition. Res. 19, 76–78. 10.1519/00124278-200502000-00013 15705049

[B22] McInnes S. E. Carlson J. S. Jones C. J. McKenna M. J. (1995). The physiological load imposed on basketball players during competition. J. Sports Sci. 13, 387–397. 10.1080/02640419508732254 8558625

[B23] Mendez-Villanueva A. Hamer P. Bishop D. (2008). Fatigue in repeated-sprint exercise is related to muscle power factors and reduced neuromuscular activity. Eur. J. Appl. Physiol. 103, 411–419. 10.1007/s00421-008-0723-9 18368419

[B24] Mero A. Komi P. V. (1987). Electromyographic activity in sprinting at speeds ranging from sub-maximal to supra-maximal. Med. Sci. Sports Exerc. 19, 266–274. 10.1249/00005768-198706000-00014 3600241

[B25] Nicolella D. P. Torres-Ronda L. Saylor K. J. Schelling X. (2018). Validity and reliability of an accelerometer-based player tracking device. PLoS ONE 13:e0191823. 10.1371/journal.pone.0191823 29420555 PMC5805236

[B26] Ortega D. R. Rodríguez Bíes E. C. La Berral de Rosa F. J. (2010). Analysis of the vertical ground reaction forces and temporal factors in the landing phase of a countermovement jump. J. Sports Sci. Med. 9, 282–287. 24149697 PMC3761745

[B27] Puente C. Abián-Vicén J. Areces F. López R. Del Coso J. (2017). Physical and physiological demands of experienced male basketball players during a competitive game. J. Strength Condition. Res. 31, 956–962. 10.1519/JSC.0000000000001577 27467516

[B28] Reina M. García-Rubio J. Pino-Ortega J. Ibáñez S. J. (2019). The acceleration and deceleration profiles of U-18 women's basketball players during competitive matches. Sports 7:E165. 10.3390/sports7070165 31284445 PMC6680831

[B29] Roecker K. Mahler H. Heyde C. Röll M. Gollhofer A. (2017). The relationship between movement speed and duration during soccer matches. PLoS ONE 12:e0181781. 10.1371/journal.pone.0181781 28742832 PMC5526535

[B30] Roell M. Mahler H. Lienhard J. Gehring D. Gollhofer A. Roecker K. (2019). Validation of wearable sensors during team sport-specific movements in indoor environments. Sensors 19:3458. 10.3390/s19163458 31394885 PMC6720677

[B31] Roell M. Roecker K. Gehring D. Mahler H. Gollhofer A. (2018). Player monitoring in indoor team sports: concurrent validity of inertial measurement units to quantify average and peak acceleration values. Front. Physiol. 9:141. 10.3389/fphys.2018.00141 29535641 PMC5835232

[B32] Scanlan A. Dascombe B. Reaburn P. (2011). A comparison of the activity demands of elite and sub-elite Australian men's basketball competition. J. Sports Sci. 29, 1153–1160. 10.1080/02640414.2011.582509 21777151

[B33] Stojanović E. Stojiljković N. Scanlan A. T. Dalbo V. J. Berkelmans D. M. Milanović Z. (2018). The activity demands and physiological responses encountered during basketball match-play: a systematic review. Sports Med. 48, 111–135. 10.1007/s40279-017-0794-z 29039018

[B34] Taylor J. B. Wright A. A. Dischiavi S. L. Townsend M. A. Marmon A. R. (2017). Activity demands during multi-directional team sports: a systematic review. Sports Med. 47, 2533–2551. 10.1007/s40279-017-0772-5 28801751

[B35] Valenti R. G. Dryanovski I. Xiao J. (2015). Keeping a good attitude: a quaternion-based orientation filter for IMUs and MARGs. Sensors 15, 19302–19330. 10.3390/s150819302 26258778 PMC4570372

[B36] Vázquez-Guerrero J. Suarez-Arrones L. Casamichana Gómez D. Rodas G. (2018). Comparing external total load, acceleration and deceleration outputs in elite basketball players across positions during match play. Kinesiology 50, 228–234. 10.26582/k.50.2.11

[B37] Whitehead S. Till K. Weaving D. Dalton-Barron N. Ireton M. Jones B. (2019). The duration-specific peak average running speeds of european super league academy rugby league match play. J. Strength Condition. Res. 10.1519/JSC.0000000000003016. [Epud ahead of print]. 30707137

[B38] Winter D. A. (2009). Biomechanics and Motor Control of Human Movement. 4th Edn. Hoboken, NJ: Wiley.

[B39] Wundersitz D. W. T. Gastin P. B. Richter C. Robertson S. J. Netto K. J. (2015). Validity of a trunk-mounted accelerometer to assess peak accelerations during walking, jogging and running. Eur. J. Sport Sci. 15, 382–390. 10.1080/17461391.2014.955131 25196466

[B40] Ziv G. Lidor R. (2009). Physical attributes, physiological characteristics, on-court performances and nutritional strategies of female and male basketball players. Sports Med. 39, 547–568. 10.2165/00007256-200939070-00003 19530751

